# Gut–Brain Connection: Deciphering Causal Pathways Between Gut Microbiota and Neuroimaging Profiles Through Mendelian Randomization

**DOI:** 10.1002/fsn3.71820

**Published:** 2026-04-26

**Authors:** Yule Zeng, Longtao Yang, Hui Liu, Hongchun Liao, Yingxia Zhou, Jun Liu

**Affiliations:** ^1^ Department of Radiology The Second Xiangya Hospital of Central South University Changsha China; ^2^ Changsha Medical University Changsha China; ^3^ Department of Surgical Operation The Second Xiangya Hospital of Central South University Changsha Hunan Province China; ^4^ Clinical Research Center for Medical Imaging in Hunan Province Changsha China; ^5^ Department of Radiology Quality Control Center in Hunan Province Changsha China

**Keywords:** brain imaging‐derived phenotypes, functional connectivity, gut microbiota, Mendelian randomization, structural connectivity

## Abstract

Recent research on the gut–brain axis (GBA) indicates that the gut microbiome can significantly influence brain structural and functional connectivity. However, the specific causal relationships between the gut microbiome and brain imaging‐derived phenotypes (IDPs) of functional/structural connectivity, as well as how the gut microbiota influences mood and cognition, remain unclear. This study utilizes data from large‐scale genome‐wide association studies (GWAS) and employs a bidirectional Mendelian randomization (MR) approach to evaluate the causal effects between the gut microbiome and brain connectivity. We obtained data on 196 gut microbiome taxa from the MiBioGen consortium and acquired IDPs for seven resting‐state networks (RSNs) from the UK Biobank (UKB). Subsequently, we conducted bidirectional MR analyses to explore their causal relationships. In the forward MR analysis, *Ruminococcus torques* , *Eubacterium fissicatena* , and *Coprobacter* exerted positive effects on the default mode network (DMN), whereas *Terrisporobacter* influenced the structural connectivity of the dorsal attention network (DAN). Conversely, Gammaproteobacteria inhibited the functional connectivity of the ventral attention network (VAN). Additionally, reverse MR analysis revealed that increased functional connectivity of the DAN was positively associated with the abundance of *Alloprevotella*. The enhanced functional connectivity of the VAN negatively impacted *Alloprevotella*, *Catenibacterium*, and Methanobacteria. Furthermore, the structural connectivity of the frontoparietal network (FPN) and somatomotor network (SMN) significantly reduced the abundance of Bacilli and *Intestinibacter*, respectively. This study utilized a bidirectional MR approach to establish causal evidence for the relationship between the gut microbiome and brain network connectivity, and support the bidirectional regulatory pattern of the GBA. These findings provide new insights into the potential roles of gut microbiota in emotional regulation, cognitive function, and neurodevelopment, and offer a theoretical basis for microbiome‐based intervention strategies.

## Graphical abstract

This graphical abstract illustrates the bidirectional causal relationships between gut microbiota and brain network connectivity identified using Mendelian randomization analysis. Genome‐wide association study data from the MiBioGen consortium (gut microbiota) and the UK Biobank (brain imaging‐derived phenotypes across seven resting‐state networks) were integrated. Forward analyses suggest that several microbial taxa may influence functional and structural connectivity within key brain networks, whereas reverse analyses indicate that brain network activity may also regulate gut microbial composition, supporting a bidirectional interaction along the gut–brain axis. The figure is created by Servier Medical Art (https://smart.servier.com/), licensed under CC BY 4.0 (https://creativecommons.org/licenses/by/4.0/). DAN, dorsal attention network; DMN, default mode network; FPN, frontoparietal network; IVs, instrumental variables; MR, Mendelian randomization; RSNs, resting‐state networks; SMN, somatomotor network; VAN, ventral attention network.

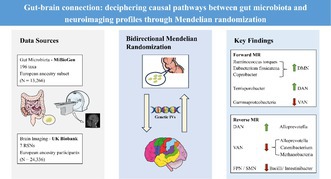

## 1 Introduction

Over the past few decades, there has been a growing interest in the intricate interactions between the gut microbiota and brain structure and function. The gut microbiota consists of trillions of highly diverse and metabolically active microbial communities residing in the gut, which play a crucial role in the bidirectional signaling processes of GBA (Cryan et al. 2019). The tripartite connection consisting of the microbiota, gut, and brain links the gastrointestinal system with the central nervous system (CNS) through various regulatory pathways including neural, endocrine, immune, and metabolic mechanisms (Gershon and Margolis 2021), and influences cognitive function, emotional regulation, and neurodegenerative processes (Socała et al. 2021).

Magnetic resonance imaging (MRI), as a non‐invasive neuroimaging technique, serves as a crucial bridge for translating our increasingly deepening understanding of the GBA into clinical applications (Montoro et al. 2022). Studies show that gut microbiota dysbiosis is associated with altered brain connectivity. For example, in amnestic mild cognitive impairment (aMCI) patients, the abundance of Bacteroidetes is negatively associated with the fractional amplitude of low‐frequency fluctuations (fALFF) value of the cerebellar vermis (Liu et al. 2021); in obese individuals, the increased relative abundance of Actinobacteria is associated with elevated fractional anisotropy (FA) in the amygdala and thalamus (Fernandez‐Real et al. 2015). Furthermore, after transplanting fecal microbiota from patients with Attention Deficit Hyperactivity Disorder (ADHD) into germ‐free (GF) mice, alterations in structural connectivity indices in brain regions such as the hippocampus and internal capsule were observed (Tengeler et al. 2020). The absence of commensal microbiota in GF mice during development also results in deficits in brain structure and function (Lu et al. 2018). However, to date, research on the causal interaction of genetic susceptibility of the brain's seven RSNs including DMN, VAN, DAN, Visual Network (VN), Limbic Network (LN), SMN, and FPN with the gut microbiota remains limited.

The gut microbiota may influence brain connectivity through various pathways. Short‐chain fatty acids (SCFAs) (Pascale et al. 2018), the primary metabolites of gut microbiota, play a significant role in the GBA. *Ruminococcus*, *Eubacterium*, *Coprobacter*, and *Terrisporobacter* are producers of SCFAs within the gut (Vital et al. 2014). Beyond improving gut health (Peng et al. 2009; Lewis et al. 2010), SCFAs can enter the CNS (Vijay and Morris 2014), and exert neuroactive properties (Stilling et al. 2016). In GF mice, colonization with SCFA‐producing strains can alleviate blood–brain barrier (BBB) permeability deficits (Braniste et al. 2014). Moreover, the gut microbiota can regulate neuroinflammation and exert neuroprotective effects through immune pathways (Patnala et al. 2017; Wang et al. 2018; Marizzoni et al. 2020). For example, in patients with end‐stage renal disease (ESRD), dysbiosis of the gut microbiota is associated with elevated pro‐inflammatory factors and abnormal functional connectivity in the DMN and the amygdala (Wang et al. 2019; Zheng et al. 2020). The gut microbiota can also synthesize or regulate neurotransmitters, such as tryptophan, gamma‐aminobutyric acid (GABA), and glutamate, thereby influencing brain network function (Strandwitz 2018; Baj et al. 2019).

The regulatory potential of the GBA has become a research hotspot in nutritional science. Dietary interventions such as supplementing probiotics, prebiotics, and functional foods are considered strategies to enhance brain function and alleviate neurological diseases (Crocetta et al. 2024; Dalile et al. 2025). Bagga et al. found that after 4 weeks of probiotic intake, healthy volunteers showed reduced activity in regions such as the precuneus, middle cingulate cortex, and parahippocampal gyrus (Bagga et al. 2019), accompanied by improvements in emotional regulation and cognitive performance (Bagga et al. 2018). Intervention with probiotic‐containing fermented dairy products in healthy women led to a significant reduction in activation intensity in the periaqueductal gray (PAG) and its associated networks, with improved emotional regulation and cognitive performance (Tillisch et al. 2013). Prebiotics refer to substrates that are selectively utilized by host microorganisms to confer a health benefit (Gibson et al. 2017), widely found in natural fruits, vegetables, and functional foods like yogurt. They primarily influence brain function through SCFAs produced after fermentation by intestinal bacteria (Torre et al. 2021). These intervention strategies may influence the functional connectivity of the DMN and attention networks by modulating the abundance of SCFA‐producers such as *Ruminococcus*, *Eubacterium*, and *Coprobacter*.

Furthermore, as a bidirectional communication system, the GBA allows brain connectivity to reverse‐regulate the composition and function of the gut microbiota. The autonomic nervous system (ANS) triggers rapid changes in the intestinal physiological environment by regulating the enteric nervous system (ENS) (Mayer and Tillisch 2011), and ENS activity is a crucial factor in shaping gut microbial composition (Rolig et al. 2017). In a rat model of intestinal ischemia, electroacupuncture can protect the intestinal barrier by activating a cholinergic anti‐inflammatory mechanism (Hu et al. 2013), and reducing intestinal permeability, potentially modulating the composition of the gut microbiota (Bonaz et al. 2018). Conversely, high‐pressure stress inhibits vagus nerve activity and disrupts gut microbial balance, participating in the pathophysiology of Irritable Bowel Syndrome (IBS) and inflammatory bowel disease (IBD) (Bonaz et al. 2018). Another important pathway is the hypothalamic–pituitary–adrenal (HPA) axis; animal studies have proven that chronic stress activation of the HPA axis has adverse effects on gut microbiota and is associated with increased IL‐6 levels (Bailey et al. 2011). Clinical evidence also confirms that psychosocial stress reduces gut microbiota diversity and hinders the colonization of beneficial bacteria (Ma et al. 2023). In summary, a tight downward regulation exists between the brain and the gut, but the specific mechanisms are not fully elucidated and require further research.

**FIGURE 1:**
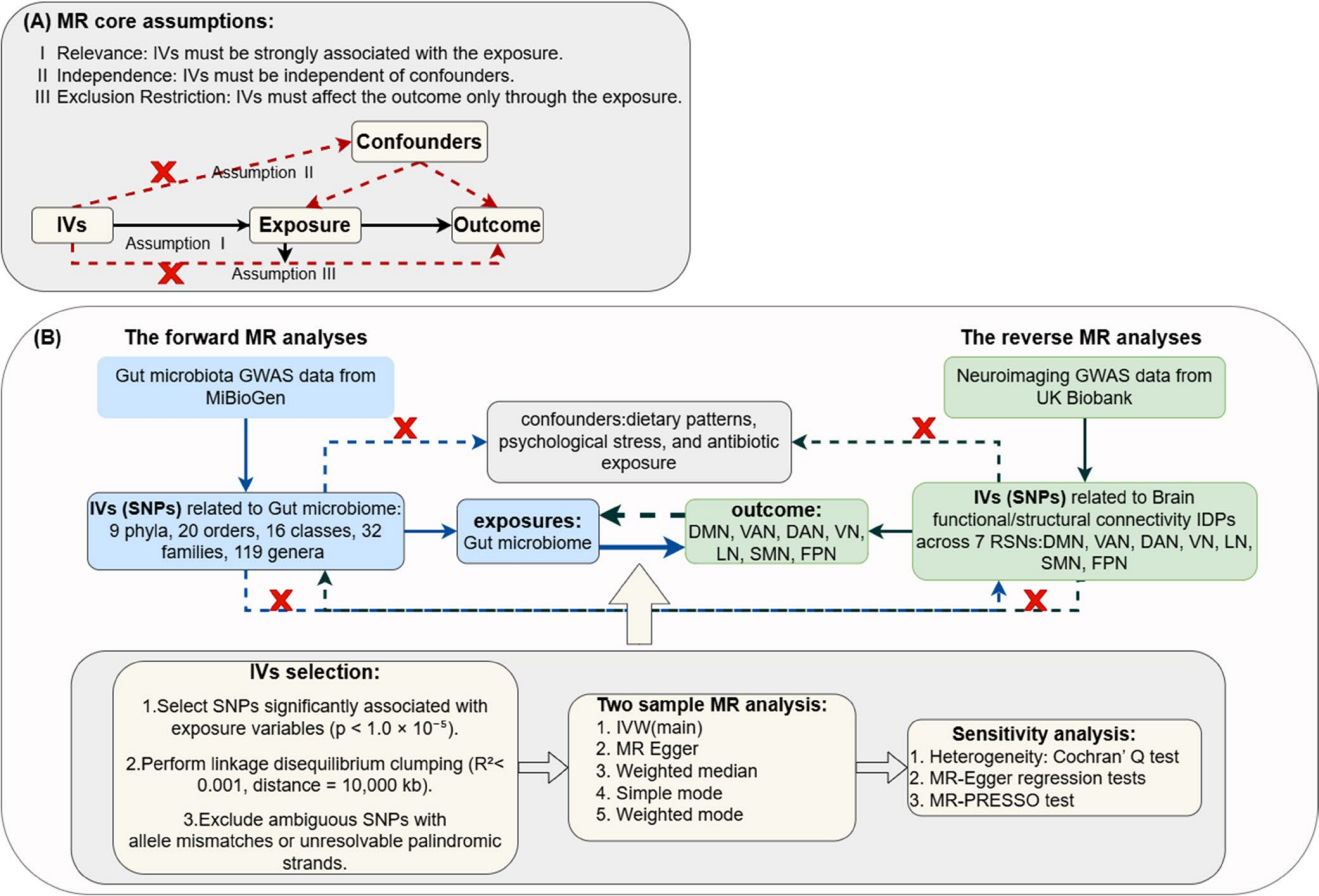
Description of the research design in this bidirectional MR study. (A) The three core assumptions of MR analyses. (B) The flowchart of the bidirectional MR study illustrates the causal association between gut microbiota and brain functional/structural connectivity. DAN, dorsal attention network; DMN, default mode network; FPN, frontoparietal network; GWAS, genome‐wide association studies; IDPs, imaging‐derived phenotypes; IVs, instrumental variables; IVW, inverse‐variance weighted; LN, limbic network; MR Egger, Mendelian randomization‐Egger; MR, Mendelian randomization; MR‐PRESSO, Mendelian randomization pleiotropy residual sum and outlier; RSNs, resting‐state networks; SMN, somatomotor network; SNPs, single nucleotide polymorphisms; VAN, ventral attention network; VN, visual network.

**FIGURE 2:**
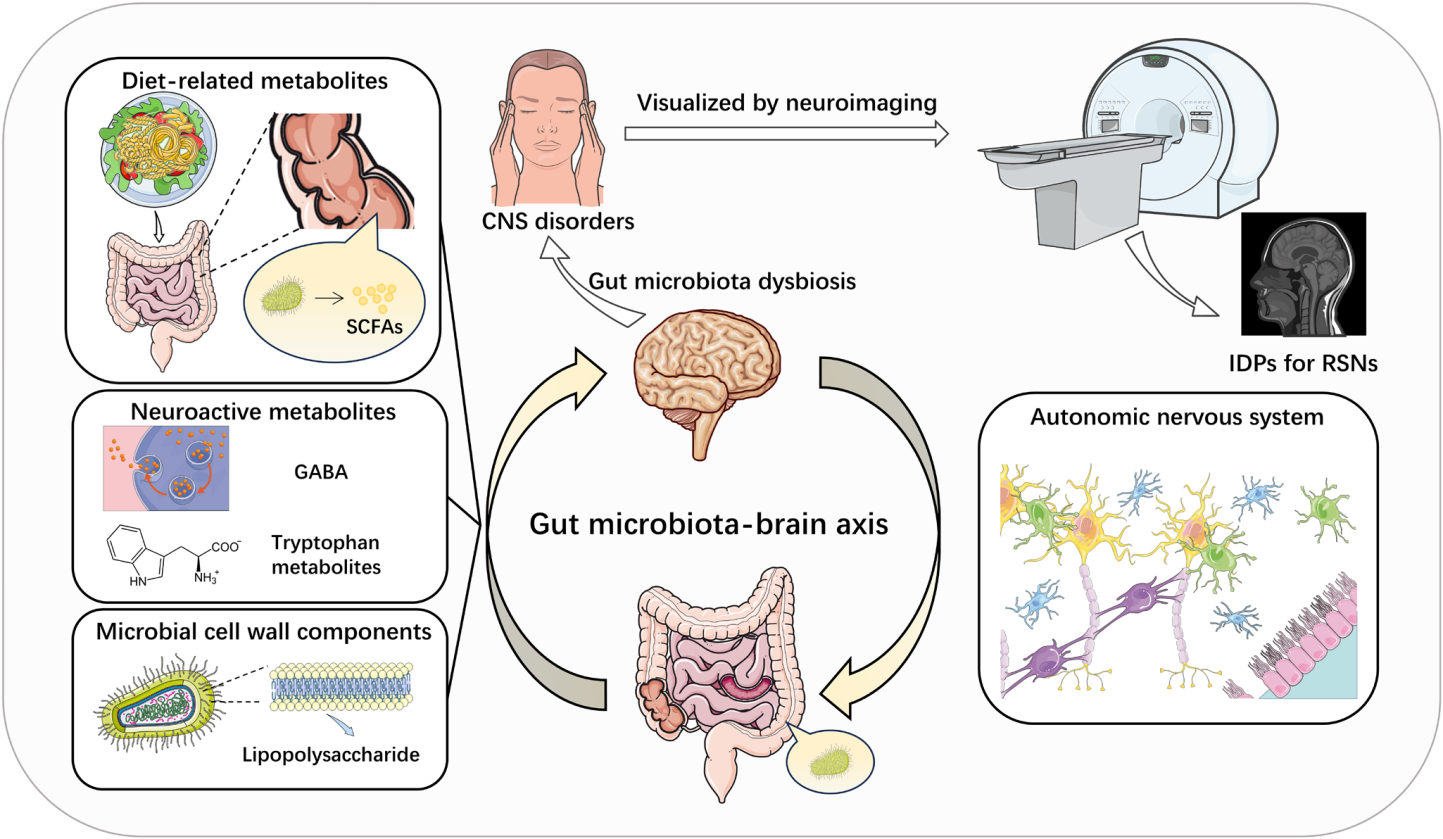
The relationship between gut microbiota and IDPs for RSNs. The gut microbiome influences the brain through various signaling pathways, including diet‐related metabolites such as SCFAs, neuroactive metabolites like GABA and tryptophan metabolites, and microbial cell wall components such as lipopolysaccharides. Dysbiosis of the gut microbiota could lead to neurological disorders, which can be observed visualized by neuroimaging. The figure is created by Servier Medical Art (https://smart.servier.com/), licensed under CC BY 4.0 (https://creativecommons.org/licenses/by/4.0/). CNS, central nervous system; GABA, gamma‐aminobutyric acid; IDPs, imaging‐derived phenotypes; RSNs, resting‐state networks; SCFAs, short‐chain fatty acids.

The GBA is jointly regulated by genetic and environmental factors (Berding et al. 2021), but previous observational studies struggle to exclude confounding factors, leaving the causal direction unclear. This study employs the MR method, using the random allocation of alleles during gamete formation as instrumental variables (IVs) to reduce interference from environmental confounding and reverse causality (Sekula et al. 2016). As shown in Figure 1A, this study strictly follows the three core assumptions of MR (Sekula et al. 2016), aiming to establish significant causal associations for the first time between gut microbiota (such as the *Ruminococcus* and *Eubacterium*) and structural/functional connectivity in key brain networks (DMN, VAN, DAN, and FPN). Integrating multi‐dimensional research evidence, this study explores the potential of gut microbiota as a neuromodulatory factor and its role in improving neurohomeostasis and cognitive health, providing a theoretical basis for microbiota‐targeted therapies for neuropsychiatric disorders. Figure 2 illustrates the associations between the gut microbiota and IDPs for seven RSNs in the brain, as well as their potential roles in central nervous system disorders.

## 2 Materials and Methods

### 2.1 Data Source

#### 2.1.1 Gut Microbiota GWAS

MiBioGen is the premier resource for human gut microbiome GWAS, amalgamating 16S ribosomal ribonucleic acid (rRNA) gene sequencing with genotypic data from 18,340 participants spanning 24 distinct cohorts (Kurilshikov et al. 2021; Huang et al. 2024). This database supplies GWAS data on the abundance levels of bacterial taxa, assessed using alpha diversity metrics (Shannon, Simpson, and Inverse Simpson) in the gut microbiota derived from microbiota quantitative trait loci (QTL) mapping analysis (MiBioGen). Cohorts included European heritage (16 cohorts, totaling 13,266 individuals), Hispanic/Latino (1 cohort, 10,097 individuals), East Asian (1 cohort, 811 individuals), Middle Eastern (1 cohort, 481 individuals), and African American (1 cohort, 114 individuals), in addition to 4 cohorts with mixed ancestries (2571 individuals). Considering differences in ancestry, age, sex, and diet, data were filtered per cohort‐specific criteria (Kurilshikov et al. 2021). To ensure ancestral homogeneity and adhere to the shared ancestry assumption of two‐sample MR, this study exclusively used the European ancestry subset (*N* = 13,266) from MiBioGen, matching the UK Biobank European‐descent outcome data.

The analysis of gut microbiota composition was conducted using V4, V3–V4, and V1–V2 variable regions of the 16S rRNA gene. Sequencing data were matched against reference databases and a log transformation was applied to correct for skewness. To include microbiome QTL mapping, only those taxa present in over 10% of the samples were considered (Kurilshikov et al. 2021; Wang, Dai, et al. 2023). After excluding 15 unidentified taxa, 196 taxa were incorporated into the MR analyses, covering 9 phyla, 20 orders, 16 classes, 32 families, and 119 genera (Kurilshikov et al. 2021). The beta estimates obtained through least squares regression reflect the impact of alleles on exposure elements.

#### 2.1.2 Neuroimaging GWAS

GWAS data related to brain functional connectivity and structural connectivity for seven resting‐state RSNs were derived from the UK Biobank (data link: https://www.fmrib.ox.ac.uk/ukbiobank/gwas_resources/index.html) (Wang, Yang, and Liu 2023). This dataset totals 24,336 participants of European ancestry and serves as the outcome dataset for bidirectional MR analysis.

### 2.2 IVs Selection

To evaluate the causal relationship between gut microbiota and brain functional/structural connectivity IDPs, the following criteria were applied for IV selection: (1) Robust association with exposure variables: The single nucleotide polymorphisms (SNPs) had to show a significant association with exposure variables, with a stringent *p*‐value threshold of less than 1.0 × 10^−5^. (2) Linkage disequilibrium verification: This step was crucial to ensure the random inheritance of genes. The clumping procedure checked for linkage disequilibrium with a squared correlation coefficient (*R* ^2^) threshold of less than 0.001 and a linkage distance set at 10,000 kilobases (kb) (Sekula et al. 2016). (3) Exclusion of ambiguous SNPs: SNPs with unclear or inconsistent allele information were excluded to maintain data integrity. Additionally, any re‐coded SNPs that could not be accurately matched were also removed. These criteria ensure the reliability of selected IVs, thus enhancing the robustness of causal inference.

### 2.3 Statistical Analysis

To confirm the reliability of our findings, we conducted two separate analyses with different IV selection strategies. The initial analysis presumed that all alleles were positioned on the forward deoxyribonucleic acid (DNA) strand without any adjustments for allele orientation. In contrast, the second, more stringent analysis took into account the strand orientations of non‐redundant SNPs and excluded all redundant SNPs. The final conclusions were based on consistent results from both analyses.

Five MR techniques were employed to evaluate causal associations between the gut microbiota and brain functional/structural connectivity‐related IDPs: inverse‐variance weighted (IVW), MR Egger regression, weighted median, simple mode, and weighted mode. IVW, as the primary method, aggregates the effect sizes of various IVs through weighted linear regression. This method offers robust causal estimates under the assumption that horizontal pleiotropy is minimal (Chen et al. 2022). The *β* estimates derived from IVW were expected to be consistent in direction with other methods. The research workflow is illustrated in Figure 1B.

We also conducted heterogeneity and pleiotropy sensitivity analyses (with all *p*‐values set to 0.1) (Wang and Zhang 2017). Cochran's *Q* test was used to evaluate the heterogeneity of IVs (Bowden and Holmes 2019). MR‐Egger regression and the MR‐PRESSO test were used to assess horizontal pleiotropy. In the MR‐Egger intercept test, the intercept was compared to zero, with a non‐zero intercept indicating the presence of horizontal pleiotropy. The MR‐PRESSO test, including global, outlier, and distortion tests, was used to identify and exclude outlier SNPs and compare causal estimates before and after removal (Verbanck et al. 2018). Finally, the *F*‐statistic (with a minimum threshold of 10) was employed to assess the strength of each IV (Sekula et al. 2016). All analyses were conducted using the two‐sample MR and MR‐PRESSO packages in R version 4.2.1.

## 3 Results

Our study utilized MR as a robust method to investigate the causal relationship between specific bacterial genera within the gut microbiome and alterations in brain structural and functional connectivity. By harnessing genetic variations as IVs, this approach enabled us to deduce causality from observational data, mitigating confounding factors commonly present in traditional epidemiological studies.

### 3.1 The Causal Influence of Gut Microbiome on Brain Connectivity

**FIGURE 3:**
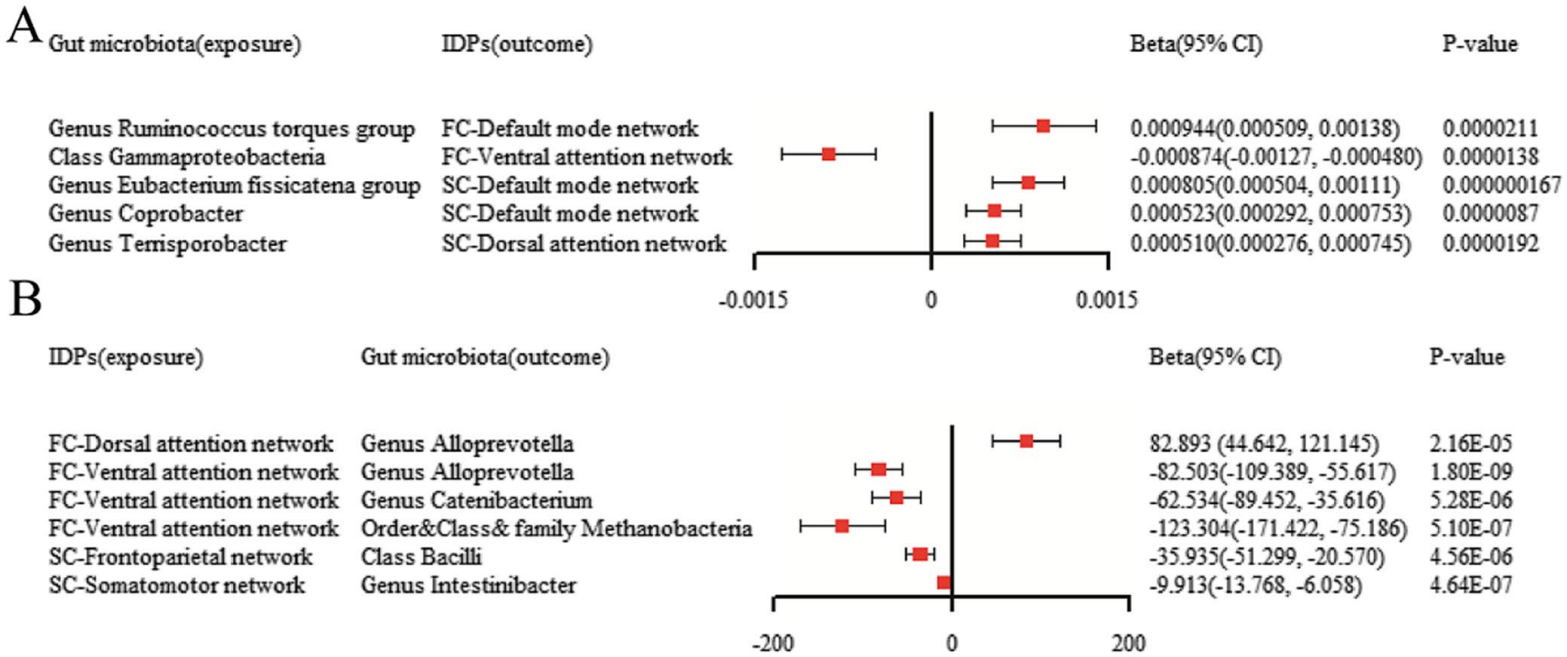
(A) Forest plots of the causal relationship between gut microbiota and brain functional/structural connectivity IDPs in the result of IVW in the forward MR analysis. (B) Forest plots of the causal relationship between brain functional/structural connectivity IDPs and gut microbiota in the result of IVW in the reverse MR analysis. FC, functional connectivity; IDPs, imaging‐derived phenotypes; SC, structural connectivity.

In the forward MR analysis, we obtained a significant causal effect (*p* < 0.05) of five gut microbiomes on brain functional/structural connectivity IDPs, as shown in Table 1 and Figure 3A. The final included MR results between each pair of exposures and outcomes met the criteria of a significant Pivw value and a consistent positive or negative sign of the *β* value for the five MR methods. The IVM method showed that the presence of the Genus *Ruminococcus torques* was significantly associated with increased functional connectivity within the DMN (*β* = 0.000944, 95% CI [0.000509, 0.00138], *p* = 2.11E‐05). The presence of both Genus *Eubacterium fissicatena* group (*β* = 0.000805, CI [0.000504, 0.00111], *p* = 1.67E‐07) and Genus *Coprobacter* (*β* = 0.000523, CI (0.000292, 0.000753), *p* = 8.70E‐06) was positively correlated with the structural connectivity of the DMN. Additionally, the Genus *Terrisporobacter* promoted the structural connectivity of DAN (*β* = 0.000510, CI (0.000276, 0.000745), *p* = 1.92E‐05). Although Class Gammaproteobacteria showed a significant negative correlation with the functional connectivity VAN (*β* = −0.000874, CI (−0.00127, −0.000480), *p* = 1.38E‐05).

**TABLE 1:** Full positive results of MR estimate for the association between gut microbiome and cerebral functional/structural connectivity.

Gut microbiome (exposure)	FC/SC IDPs (outcome)	MR method	No. SNP	*F*	*β* (95% CI)	*p*
Genus *Ruminococcus torques* group	FC‐default mode network	IVW (MRE)	9	19.3	0.000944 (0.000509, 0.00138)	2.11E‐05
		MR Egger	9		0.00174 (−0.000567, 0.00404)	
		Weighted median	9		0.000948 (−0.000149, 0.00205)	
		Simple mode	9		0.00134 (−0.000203, 0.00288)	
		Weighted mode	9		0.00117 (−0.000385, 0.00273)	
Class Gammaproteobacteria	FC‐ventral attention network	IVW (MRE)	7	19.68	−0.000874 (−0.00127, −0.000480)	1.38E‐05
		MR Egger	7		−0.000574 (−0.00276, 0.00161)	
		Weighted median	7		−0.000706 (−0.00156, 0.000149)	
		Simple mode	7		−0.000711 (−0.00196, 0.000542)	
		Weighted mode	7		−0.000650 (−0.00190, 0.000602)	
Genus *Eubacterium fissicatena* group	SC‐default mode network	IVW (MRE)	7	19.44	0.000805 (0.000504, 0.00111)	1.67E‐07
		MR Egger	7		0.000488 (−0.00252, 0.00350)	
		Weighted median	7		0.000925 (0.000155, 0.00169)	
		Simple mode	7		0.00100 (−6.277E‐05, 0.00207)	
		Weighted mode	7		0.00101 (−0.000180, 0.00220)	
Genus *Coprobacter*	SC‐default mode network	IVW (MRE)	10	19.42	0.000523 (0.000292, 0.000753)	8.70E‐06
		MR Egger	10		0.000984 (−0.00108, 0.00305)	
		Weighted median	10		0.000610 (−0.000193, 0.00141)	
		Simple mode	10		0.000717 (−0.000537, 0.00197)	
		Weighted mode	10		0.000762 (−0.000388, 0.00191)	
Genus *Terrisporobacter*	SC‐dorsal attention network	IVW (MRE)	5	19.72	0.000510 (0.000276, 0.000745)	1.92E‐05
		MR Egger	5		0.000289 (−0.00180, 0.00238)	
		Weighted median	5		0.000399 (−0.000494, 0.00129)	
		Simple mode	5		0.000392 (−0.000833, 0.00162)	
		Weighted mode	5		0.000395 (−0.000797, 0.00159)	

Abbreviations: CI, confidence interval; IDPs, imaging‐derived phenotype; IVW, inverse variance weighted; MR Egger, Mendelian randomization‐Egger; MR, Mendelian randomization; MRE, multiplicative random effects model; QSM, quantitative susceptibility mapping; SNP, single nucleotide polymorphism.

### 3.2 The Causal Influence of Brain Connectivity on Gut Microbiome

To further explore the causal relationship between specific gut microbiome and changes in brain structural–functional connectivity, we performed inverse MR analysis, and the significance results are shown in Table 2 and Figure 3B. Our study identified significant causal effects of four brain structural–functional connectivity networks on intestinal flora (*p* < 0.05). The functional connectivity of DAN had a promoting effect on Genus *Alloprevotella* (*β* = 82.893, CI = [44.642, 121.145], *p* = 2.16E‐05). However, the functional connectivity on VAN could be negatively correlated with three specific gut microbiomes: Genus *Alloprevotella* (*β* = −82.503, CI = [−109.389, −55.617], *p* = 1.80E‐09), Genus *Catenibacterium* (*β* = −62.534, CI = [−89.452, −35.616], *p* = 5.28E‐06), and Order & Class & family Methanobacteria (*β* = −123.304, CI = [−171.422, −75.186], *p* = 5.09825E‐07). In addition, enhanced structural connectivity of the FPN and structural connectivity of SMN inhibited the expression of intestinal Class Bacilli (*β* = −35.935, CI = [−51.299, −20.570], *p* = 4.56E‐06) and Genus *Intestinibacter* (*β* = −9.913, CI = [−13.768, −6.058], *p* = 4.64E‐07). Similarly, the results of the other complementary MR methods were in the same direction as the above IVW methods.

**TABLE 2:** Full positive results of inverse MR estimate for the association between gut microbiome and cerebral functional/structural connectivity.

FC/SC IDPs (exposure)	Gut microbiome (outcome)	MR method	No. SNP	*F*	*β* (95% CI)	*p*
FC‐dorsal attention network	Genus *Alloprevotella*	IVW (MRE)	9	19.60	82.893 (44.642, 121.145)	2.16E‐05
		MR Egger	9		202.265 (−403.589, 808.118)	
		Weighted median	9		77.853 (−12.645, 168.351)	
		Simple mode	9		77.871 (−65.474, 221.216)	
		Weighted mode	9		75.088 (−59.295, 209.471)	
FC‐ventral attention network	Genus *Alloprevotella*	IVW (MRE)	4	19.53	−82.503 (−109.389, −55.617)	1.80E‐09
		MR Egger	4		−64.517 (−993.943, 864.909)	
		Weighted median	4		−73.894 (−207.897, 60.108)	
		Simple mode	4		−68.002 (−237.100, 101.095)	
		Weighted mode	4		−67.278 (−237.540, 102.983)	
FC‐ventral attention network	Genus *Catenibacterium*	IVW (MRE)	5	19.53	−62.534 (−89.452, −35.616)	5.28E‐06
		MR Egger	5		−242.473 (−906.306, 421.360)	
		Weighted median	5		−54.0991 (−169.568, 61.369)	
		Simple mode	5		−41.129 (−191.733, 109.474)	
		Weighted mode	5		−41.860 (−176.247, 92.527)	
FC‐ventral attention network	Order & class & family Methanobacteria	IVW (MRE)	6	19.53	−123.304 (−171.422, −75.186)	5.09825E‐07
		MR Egger	6		−304.843 (−867.603, 257.918)	
		Weighted median	6		−119.668 (−225.041, −14.294)	
		Simple mode	6		−118.068 (−261.339, 25.203)	
		Weighted mode	6		−118.068 (−262.344, 26.208)	
SC‐frontoparietal network	Class Bacilli	IVW (MRE)	16	19.99	−35.935 (−51.299, −20.570)	4.56E‐06
		MR Egger	16		−18.537 (−143.658, 106.583)	
		Weighted median	16		−34.027 (−67.642, −0.4123)	
		Simple mode	16		−62.402 (−117.764, −7.040)	
		Weighted mode	16		−62.402 (−119.673, −5.131)	
SC‐somatomotor network	Genus *Intestinibacter*	IVW (MRE)	13	20.12	−9.913 (−13.768, −6.058)	4.64E‐07
		MR Egger	13		−16.184 (−69.537, 37.168)	
		Weighted median	13		−8.762 (−19.021, 1.496)	
		Simple mode	13		−7.816 (−24.953, 9.320)	
		Weighted mode	13		−7.632 (−24.214, 8.949)	

Abbreviations: CI, confidence interval; IDPs, imaging‐derived phenotype; IVW, inverse variance weighted; MR Egger, Mendelian randomization‐Egger; MR, Mendelian randomization; MRE, multiplicative random effects model; QSM, quantitative susceptibility mapping; SNP, single nucleotide polymorphism.

### 3.3 Sensitivity Analyses

Several sensitivity analyses were performed to ensure the robustness of the findings (Table 3). There was no heterogeneity in the significant causal associations we obtained in either forward or reverse MR analyses, and the respective results were not affected by horizontal pleiotropy (all *p* > 0.1). Thus, the results of the sensitivity analyses further demonstrate the reliability of our MR analyses.

**TABLE 3:** Sensitivity analyses for association between gut microbiome and cerebral functional/structural connectivity.

Gut microbiome (exposure)	FC/SC IDPs (outcome)	No. SNP	Pleiotropy		Heterogeneity	
			MR‐PRESSO global *p*	MR‐Egger *p*	IVW test *p*	MR‐Egger *p*
Genus *Ruminococcus torques* group	FC‐default mode network	9	0.688	0.490	0.980	0.982
Class Gammaproteobacteria	FC‐ventral attention network	7	0.894	0.789	0.903	0.836
Genus *Eubacterium fissicatena* group	SC‐default mode network	7	0.961	0.841	0.961	0.920
Genus *Coprobacter*	SC‐default mode network	10	0.840	0.658	0.999	0.998
Genus *Terrisporobacter*	SC‐dorsal attention network	5	0.414	0.838	0.984	0.955

FC/SC IDPs (exposure)	Gut microbiome (outcome)	No. SNP	Pleiotropy		Heterogeneity	
			MR‐PRESSO global *p*	MR‐Egger *p*	MR‐PRESSO global *p*	MR‐Egger *p*
FC‐dorsal attention network	Genus *Alloprevotella*	9	0.980	0.709	0.968	0.948
FC‐ventral attention network	Genus *Alloprevotella*	4	0.982	0.973	0.981	0.916
FC‐ventral attention network	Genus *Catenibacterium*	5	0.995	0.629	0.989	0.999
FC‐ventral attention network	Order & class & family Methanobacteria	6	0.226	0.557	0.894	0.869
SC‐frontoparietal network	Class Bacilli	16	0.914	0.792	0.983	0.974
SC‐somatomotor network	Genus *Intestinibacter*	13	0.973	0.820	0.998	0.995

Abbreviations: IDPs, imaging‐derived phenotype; IVW, inverse variance weighted; MR‐Egger, Mendelian randomization‐Egger; MR‐PRESSO, Mendelian Randomization Pleiotropy RESidual Sum and Outlier.

## 4 Discussion

With the rapid progress in neuroimaging and microbiome research, the perspective that the gut microbiota regulates brain structure and function via the GBA has been widely recognized, but the causal direction remains to be elucidated. This research utilizes bidirectional MR methods to confirm the causal link between gut microbiota and brain connectivity from a genetic standpoint. The results show that in the forward MR analysis, *R. torques* , *E. fissicatena* , and the *Coprobacter* genus significantly enhanced DMN connectivity. *Terrisporobacter* and Gammaproteobacteria exerted positive and negative effects on the connectivity of the DAN and VAN, respectively. Reverse MR reveals that brain network connectivity reciprocally regulates microbial abundance: increased functional connectivity of the DAN was positively correlated with *Alloprevotella* abundance, whereas enhanced VAN functional connectivity had negative impacts on *Alloprevotella*, *Catenibacterium*, and Methanobacteria. Furthermore, the structural connectivity of the FPN and SMN significantly reduced the abundance of Bacilli and *Intestinibacter*. These findings support the bidirectional regulatory mechanisms of the GBA.

In positive MR analysis, we identified that *R. torques* , *E. fissicatena* , and the *Coprobacter* genus all have significant positive effects on DMN connectivity, suggesting a synergistic role in emotional regulation and the maintenance of cognitive function. As a core brain network closely related to self‐reference, emotional rumination, and episodic memory, abnormal DMN connectivity has been confirmed in various diseases such as IBS, chronic insomnia (CI), and bipolar disorder (BD), which are generally accompanied by gut dysbiosis, suggesting the potential role of the GBA in their pathogenesis (Osadchiy et al. 2020; Li et al. 2022; Feng et al. 2022). Previous studies have shown that the abundance of the Ruminococcaceae family is significantly reduced in IBS patients and is associated with increased anxiety symptoms (Yang et al. 2023; Yusof et al. 2017). As an important member of this family, *R. torques* abundance is positively correlated with the functional connectivity variability between multiple networks such as the cognitive control network (CCN)–DMN. Changes in the abundance of the *Eubacterium* and *Coprobacter* genera have also been reported to be associated with certain brain network indices. Furthermore, *Coprobacter* abundance is related to the functional integrity of the left angular gyrus (LAG), a key hub of the DMN, whereas reduced regional homogeneity (ReHo) in the LAG is associated with impaired executive function and attention in CI patients (Feng et al. 2022). These lines of evidence suggest that these microbiota may participate in maintaining DMN network homeostasis, thereby affecting emotional and cognitive regulation.

At the mechanistic level, most of these microbiota are SCFA producers (Vital et al. 2014), and their metabolites can participate in the regulation of brain network homeostasis by modulating blood–brain barrier integrity, neuroinflammation, and neurotransmitter metabolism (Wang et al. 2019; Zheng et al. 2020; Li et al. 2022; O'Riordan et al. 2022). For example, *Ruminococcus* may participate in the bidirectional communication of the GBA by promoting serotonin release, a mechanism that may be impaired in IBS (Labus et al. 2019). In BD patients, gut dysbiosis is accompanied by a decreased capacity to synthesize neuroactive metabolites and weakened limbic–cortical functional connectivity, further suggesting that microbial metabolic abnormalities may be involved in brain network changes (Li et al. 2022). Therefore, the genetic causality revealed in this study provides a theoretical basis for intervention strategies based on diet and microbiome regulation, suggesting that adjusting relevant microbiota structures through dietary fiber intake, prebiotic supplementation, and fermented foods may help improve brain network‐related emotional and cognitive abnormalities. Existing clinical studies provide indirect support: treatment with the probiotic *Bifidobacterium longum* NCC3001 (BL) can alleviate depressive symptoms in IBS patients and reduce the response of brain regions such as the fronto‐limbic areas to negative emotional stimuli (Pinto‐Sanchez et al. 2017).

Notably, our MR results show the differential impacts of different gut microbiota on the two attention network systems: the SCFA‐producing genus *Terrisporobacter* has a positive promoting effect on DAN structural connectivity, whereas the pro‐inflammatory Gammaproteobacteria class, containing lipopolysaccharide (LPS), inhibits the functional connectivity of the VAN. Previous clinical studies provide indirect support; for example, *Terrisporobacter* is significantly reduced in H‐type hypertensive ischemic stroke patients, and its abundance is related to the degree of neurological recovery (Yu et al. 2023); conversely, the LPS components of Gammaproteobacteria can induce the release of pro‐inflammatory factors and trigger neuroinflammatory responses (Oyama et al. 2004; Rothhammer et al. 2016), and its abundance tends to increase in cognitive impairment‐related diseases such as post‐stroke cognitive impairment and Alzheimer's disease (Ling et al. 2020; Liu et al. 2019). As complementary functional attention systems, the DAN and VAN are responsible for top‐down attention control and bottom‐up attention capture, respectively (Alves et al. 2022). Their coordination imbalance has been confirmed in diseases such as major depressive disorder and ADHD, and is associated with abnormal emotional regulation and attention function deficits (Wu et al. 2023).

Crucially, the genetic findings of this study are supported by direct causal evidence from animal models based on human samples. Guzzardi et al. (2023) found that transplanting fecal microbiota from children with high cognitive performance into germ‐free mice resulted in superior learning and memory abilities in the recipient mice during adulthood, with relevant microbiota including members of the *Ruminococcus* and *Eubacterium* genera, suggesting that microbiota may participate in the phenotype transfer related to cognitive development. Meanwhile, Tengeler et al. (2020) found that transplanting the microbiota of ADHD patients led to abnormal brain functional connectivity and anxiety‐like behavior in recipient mice, and observed a negative correlation between *Eubacterium* abundance and anxiety severity, further corroborating the direct regulatory role of human‐derived microbiota differences on attention‐related brain networks. These cross‐species experiments from human donors to animal recipients provide evidence for microbiota–brain phenotype transfer, consistent with the MR inference direction of this study, supporting the causal chain from genetic association to biological evidence. Moreover, gut intervention strategies based on nutritional regulation (such as promoting SCFA producers and inhibiting pro‐inflammatory microbiota) may have potential application value in brain network development and long‐term cognitive protection (Onofrj et al. 2022). However, the specific neurobiological mechanisms still require further verification through longitudinal and experimental studies.

Reverse MR analysis revealed the causal regulatory effect of specific brain networks on the gut microbiota, a novel finding that highlights the importance of top‐down regulatory pathways in the GBA. We speculate that brain network activity may affect the gut physiological state through the autonomic nervous system and neuroendocrine pathways, including regulating intestinal peristalsis, secretion, and immune signaling (Breit et al. 2018), thereby altering the physicochemical environment of the gut and shaping microbial niches (Bonaz et al. 2016). Enhanced DAN functional connectivity may inhibit the release of pro‐inflammatory factors such as TNF by activating the vagus nerve and its mediated cholinergic anti‐inflammatory pathway, thereby reducing intestinal inflammation and maintaining mucosal homeostasis (Pavlov and Tracey 2005), providing a more favorable environment for the *Alloprevotella* genus. In contrast, when the VAN, FPN, and SMN are over‐activated, it may be accompanied by enhanced HPA axis stress response and up‐regulated sympathetic nerve activity, promoting glucocorticoid release. This process may negatively impact microorganisms such as *Alloprevotella* and Methanobacteria by interfering with gut barrier function and local immune balance. Previous studies have shown that psychological stress can significantly affect gut microbial composition (Bailey et al. 2011; Bharwani et al. 2016; De Palma et al. 2015) and induce intestinal inflammatory responses (Sun et al. 2013). Furthermore, the opposite regulatory directions of the DAN and VAN toward the *Alloprevotella* genus also suggest that different attention networks may play different roles in maintaining gut micro‐ecological homeostasis. Although these hypotheses based on neuroanatomy and physiology have certain biological plausibility, future research can combine technologies such as optogenetics or chemogenetics to systematically explore how specific neural network activity regulates the evolution of gut micro‐ecology through neuro‐endocrine‐immune pathways, which will help develop potential brain‐derived microbial intervention strategies for neuropsychiatric diseases.

The potential causal relationships identified in this study between the gut microbiota and brain network connectivity provide important insights for nutritional intervention strategies. Studies have shown that diet is a key factor in regulating the composition and metabolic activity of the gut microbiota (Li et al. 2025; Borrego‐Ruiz and Borrego 2025). For example, foods rich in dietary fiber, whole grains, fruits, and vegetables can alter the gut micro‐ecological structure by promoting the growth of SCFA‐producing bacteria (Crocetta et al. 2024), whereas fermented foods and probiotic supplements can directly regulate the abundance of specific microbial taxa. These diet‐driven microbial changes may further affect brain functional connectivity patterns through the GBA, thus having potential impacts on cognitive function, emotional regulation, and neurodevelopment. Combining the MR results of this study, certain gut genera related to SCFA production may play a role in regulating the structural or functional connectivity of key brain networks. Therefore, promoting the growth of these beneficial microbiota through nutritional intervention means such as dietary fiber, prebiotics, or fermented foods may provide a feasible dietary strategy for improving neurohomeostasis and supporting cognitive health, especially in populations with diseases related to GBA dysfunction.

This study has several advantages. First, bidirectional MR effectively controls for confounding factors and reverse causality common in observational studies. Second, multiple MR methods and sensitivity analyses improve the robustness of the results, mapping brain network features that may be affected by the microbiome. These findings suggest that the gut microbiome may become a potential target for neuropsychiatric interventions. Meanwhile, brain functional abnormalities may in turn affect gut microbial structure, suggesting that improving brain network function through neuromodulation techniques such as brain stimulation therapies (Arulchelvan and Vanneste 2025) may indirectly restore gut micro‐ecological balance. However, this study still has certain limitations. First, the lowest taxonomic level of exposure data are at the genus level, limiting analysis at the species level. Second, human gut microbial composition is affected by environment and lifestyle, which may still lead to some genetic heterogeneity. Furthermore, potential pleiotropy may still exist among some microorganisms. Finally, although MR provides evidence for causal inference, the specific molecular mechanisms still need further verification through animal experiments and clinical studies. Future research can verify these associations in larger samples and different populations, and combine multi‐omics and longitudinal studies to further clarify the molecular mechanisms of the GBA and promote the translational application of microbiome‐based intervention strategies in neurological diseases.

## 5 Conclusion

This study evaluates the potential causal relationship between the gut microbiome and brain network connectivity from a genetic perspective through bidirectional MR analysis. The results show that specific gut microbiota can influence the connectivity of different brain networks, whereas brain network activity may also reciprocally regulate gut microbial composition, supporting the bidirectional regulatory mode of the GBA. These findings not only expand our understanding of the mechanisms underlying gut microbiota–brain network interactions but also provide new research perspectives for explaining emotional disorders, cognitive dysfunction, and neurodevelopmental diseases associated with gut–brain imbalance. Meanwhile, this study suggests that targeting gut micro‐ecology and brain network function through strategies such as dietary intervention, probiotic supplementation, or neuromodulation may become potential pathways for intervening in neuropsychiatric disorders. Future research can combine multimodal neuroimaging and multi‐omics approaches to further elucidate the molecular mechanisms of microbiome–brain network interactions, thereby promoting the application of GBA‐based precision intervention strategies in the prevention and treatment of neurological diseases.

## Author Contributions

**Hongchun Liao:** software. **Hui Liu:** methodology. **Longtao Yang:** formal analysis. **Yule Zeng:** writing – original draft. **Jun Liu:** writing – review and editing. **Yingxia Zhou:** supervision.

## Conflicts of Interest

The authors declare no conflicts of interest.

## Data Availability

The data that support the findings of this study are available from the corresponding author upon reasonable request.
